# Surgical Antibiotic Prophylaxis Utilization and Predictors of Surgical Site Infections at Debre Tabor Comprehensive Specialized Hospital, Northwest Ethiopia: Prospective Cohort Study

**DOI:** 10.1155/bmri/6612112

**Published:** 2026-02-24

**Authors:** Yared Andargie Ferede, Muluken Adela Alemu, Samuel Berihun Dagnew, Tilaye Arega Moges, Achenef Bogale Kassie, Woretaw Sisay Zewdu, Andebet Birhan Mebratie

**Affiliations:** ^1^ Pharmacology Unit, Department of Pharmacy, College of Health Sciences, Debre Tabor University, Debre Tabor, Ethiopia, dtu.edu.et; ^2^ Department of Clinical Pharmacy, College of Health Sciences, Debre Tabor University, Debre Tabor, Ethiopia, dtu.edu.et; ^3^ Department of Pharmacy, College of Health Sciences, Debre Tabor University, Debre Tabor, Ethiopia, dtu.edu.et

**Keywords:** antibiotic prophylaxis, Debre Tabor Comprehensive Specialized Hospital, Ethiopia, surgical site infection

## Abstract

**Background:**

Surgical site infections (SSIs) are a major postoperative complication, particularly in resource‐limited settings such as Ethiopia. Despite evidence‐based guidelines, inappropriate surgical antibiotic prophylaxis (SAP) remains common. This study is aimed at assessing SAP utilization patterns, SSI prevalence, and associated factors at Debre Tabor Comprehensive Specialized Hospital.

**Methods:**

A prospective cohort study was conducted among 342 surgical patients from February 1 to May 30, 2025. Adherence of SAP was assessed based on standard national and international guidelines. Descriptive, bivariate, and multivariable analyses were conducted using IBM SPSS software Version 27. Statistical significance was set at *p* < 0.05.

**Results:**

Preoperative prophylaxis was administered to 69.3% of patients, although 83.1% received inappropriate antibiotics. Ceftriaxone (50.2%) and its combination with metronidazole (27.4%) were the most commonly used antibiotics. The overall magnitude of SSI was 10.8% (95% CI: 7.5–14.1). Elective surgery (AOR = 0.57), absence of comorbidities (AOR = 0.13), and appropriate SAP use (AOR = 0.67) were associated with reduced odds of developing SSI. In contrast, procedures lasting 2–3 h (AOR = 4.21) or more than 3 h (AOR = 5.26), contaminated (AOR = 3.42) or dirty wounds (AOR = 9.35), antibiotic prophylaxis administered more than 1–2 h before incision (AOR = 5.34), and postoperative SAP duration exceeding 72 h (AOR = 5.63) were linked to an increased risk of SSIs.

**Conclusion:**

SSIs are still a significant burden at Debre Tabor Comprehensive Specialized Hospital. Strengthening adherence to SAP guidelines is essential to reduce infections and improve patient outcomes.

## 1. Introduction

Surgical procedures, although often lifesaving, carry a significant risk of infection due to the exposure of sterile tissues to external pathogens [1]. To mitigate this risk, antibiotics are commonly used as surgical prophylaxis [2]. Surgical antibiotic prophylaxis (SAP) involves the administration of a short course of an antimicrobial agent prior to surgery to prevent postoperative infections [3]. Proper use of SAP is crucial to patient outcomes, particularly in reducing surgical site infections (SSIs), which are among the most common complications following surgery [4]. In addition to preoperative prophylaxis, antibiotics may also be administered postoperatively when an infection is suspected or confirmed [5]. Postoperative antibiotic therapy is aimed at treating or preventing infections, particularly in high‐risk surgeries where the likelihood of infection is elevated. However, prolonged or inappropriate use of antibiotics can lead to complications, including the emergence of multidrug‐resistant organisms (MDROs), extended hospital stays, and increased healthcare costs [6].

SSIs are defined as infections occurring at or near the surgical incision within 30 days postoperatively, or within 90 days if an implant is placed [7]. They are among the most common healthcare‐associated infections in surgical patients, ranking second among hospital‐acquired infections. SSIs account for approximately 14%–17% of all hospital‐acquired infections and 38% of infections in surgical patients [8, 9]. In low‐ and middle‐income countries (LMICs), SSI rates range from 8% to 30% of surgical procedures, representing the most frequent hospital‐acquired infection and contributing substantially to morbidity, mortality, and economic burden [10].

Despite the widespread use of antibiotics in surgical settings, there is growing concern about their inappropriate use, which contributes to the global problem of antimicrobial resistance [11]. Nearly 30%–50% of antimicrobials used in hospitals are prescribed for surgical prophylaxis [12]. Overprescription, inadequate dosing, and prolonged use beyond the recommended duration are major factors driving the emergence of resistant pathogens, reducing the effectiveness of standard treatments [13].

SAP plays a critical role in preventing SSIs, which are among the most common postoperative complications [14]. However, improper use such as unnecessary administration, incorrect antibiotic selection, inappropriate timing, and prolonged duration remains a significant concern [15]. Studies indicate that 30%–90% of SAP prescriptions are inappropriate [16]. Postoperative wound infections occur in approximately 15% of elective surgeries and up to 30% of contaminated or dirty procedures [17]. Globally, the pooled incidence of SSIs is reported to be 2.5% [18], whereas a meta‐analysis in sub‐Saharan Africa reported higher rates, ranging from 6.8% to 26% [19].

Despite global and national efforts to reduce SSIs through evidence‐based guidelines, including appropriate SAP, significant challenges persist in resource‐limited settings such as Ethiopia [20]. Few studies have addressed this issue in these contexts, and none have been conducted in the current study setting. Therefore, this study is aimed at assessing the patterns of prophylactic antibiotic use, the magnitude of SSIs, and their associated predictors in the surgical ward of Debre Tabor Comprehensive Specialized Hospital (DTCSH). The findings are intended to identify gaps in SAP practices, provide local evidence to guide hospital policies, and serve as a baseline for future interventions, ultimately contributing to improved SSI control and the appropriate use of SAP.

## 2. Methods

### 2.1. Study Area

The study was conducted in the surgical wards of DTCSH, located in Debre Tabor Town, South Gondar Zone, Amhara Region, Northwest Ethiopia. The hospital serves as a referral and teaching institution, offering specialized services including surgery, internal medicine, pediatrics, and obstetrics and gynecology. The surgery department comprises the major surgical ward, orthopedic ward, and gynecology and obstetrics ward.

### 2.2. Study Design and Period

A prospective cohort study was conducted among patients undergoing surgery at DTCSH from February 1 to May 30, 2025. Eligible patients were enrolled at the time of their surgical procedure and followed for 30 days, or up to 90 days for implant procedures, to determine the occurrence of SSIs. SAP was administered before surgery and recorded at baseline. The outcome (SSI) was assessed during hospitalization and throughout the postoperative follow‐up period, ensuring a clear temporal relationship between exposure and outcome.

### 2.3. Source Population

The source population comprised all patients admitted for surgical procedures at DTCSH during the study period.

### 2.4. Study Population

The study population included patients admitted to the surgical wards of DTCSH during the study period who met the inclusion criteria.

### 2.5. Inclusion Criteria

All adult patients (≥ 18 years) admitted to DTCSH for major surgical procedures during the study period with a postoperative hospital stay of at least 48 h were eligible for inclusion.

### 2.6. Exclusion Criteria

Patients who underwent minor surgical procedures, were operated on elsewhere and later referred to DTCSH, had preexisting SSIs, or had missing essential information were excluded from the study.

### 2.7. Sample Size Determination and Sampling Techniques

The sample size was determined using the single population proportion formula with a 95% confidence level (*Z* = 1.96), a margin of error of 5% (*E*), and an estimated population proportion (*P*) of 0.5:$\begin{array}{cc} \; & \text{n}=\frac{Z^{2}P\left(1-P\right)}{E^{2}} \end{array}$

Accordingly, the initial sample size was calculated as follows:$\begin{array}{cc} \; & \text{n}=\frac{{\left(1.96\right)}^{2}\times 0.5\times \left(10.5-\right)}{{\left(0.05\right)}^{2}}=384.16384\approx \end{array}$

Since the total number of patients admitted to the surgical wards over the preceding 4 months was fewer than 10,000 (*N* = 1640), a finite population correction formula was applied:$\begin{array}{cc} \; & \text{nf}=\text{n}/\left(1+\frac{\text{n}}{\text{N}}\right)=384311/\left(1+\frac{384}{1640}\right)= \end{array}$ where *n* *f* is adjusted sample size, *n* is initial sample size, and *N* is total population size.

To account for potential incomplete data and response errors, 10% was added, resulting in a final sample size of 342 participants. Consecutive sampling was employed, including all eligible surgical patients admitted during the study period until the required sample size was achieved.

### 2.8. Study Variables

The primary outcome variable in this study was the occurrence of SSI. Independent variables included sociodemographic characteristics (age, sex, and marital status), clinical factors (presence of comorbidities, duration of operation, type of admission, and wound classification), and antibiotic‐related variables (use and choice of prophylactic antibiotics, timing of administration, dosage regimen, and postoperative antibiotic use duration).

### 2.9. Data Collection Tool and Procedures

Data collection tools were developed with minor adaptations from the American Society of Health‐System Pharmacists (ASHP) guidelines, World Health Organization (WHO) and Centers for Disease Control and Prevention (CDC) recommendations, and relevant previously published studies [21–23]. The instrument included patient sociodemographic and clinical characteristics, details of antibiotic prophylaxis (drug name, dose, route, timing, and duration), and the occurrence of SSIs during hospitalization. The tool was formatted and deployed using Kobo Toolbox to facilitate efficient and standardized digital data collection.

Sociodemographic data were collected through direct patient interviews, whereas clinical and other relevant information were extracted from medical charts. Postoperative wound assessments were conducted daily during hospitalization using a structured checklist adapted from the CDC SSI surveillance tool. These assessments were performed by trained operating room nurses, and any suspected SSI was confirmed by the attending surgeon. Assessment criteria included clinical signs such as purulent discharge, localized pain, redness, swelling, increased warmth, and, when available, microbiological evidence.

After discharge, patients were followed for 30 days, and up to 90 days for implant‐related procedures, in accordance with CDC recommendations. Postdischarge follow‐up was conducted through scheduled outpatient clinic visits and telephone interviews for patients unable to return. All data were collected by two trained nurses under the close supervision of the principal investigator. The ASHP guideline, WHO recommendations, and national standard treatment guidelines were used as benchmarks to evaluate the rational use of SAP.

### 2.10. Data Management

The study was conducted prospectively, and all participants were followed from enrollment to study completion. All study variables were recorded in real time using standardized data collection forms. Immediate verification and monitoring of the forms ensured that no data were missing for any participant. Therefore, no imputation or other methods for handling missing data were required.

### 2.11. Data Quality Assurance and Bias Control

Before data collection, the tool was reviewed by three academic experts in pharmacy, public health, and infectious diseases to ensure content validity. A pretest was conducted at Woldia Comprehensive Specialized Hospital using 5% of the study population to identify ambiguous or unclear items, which were subsequently rephrased for clarity. Participants included in the pretest were excluded from the main study, and their data were not included in the final analysis to avoid contamination. Data collectors were trained on standardized procedures, and data were reviewed daily by the principal investigator for completeness and consistency. Records with missing key outcome variables were excluded from the analysis. Potential sources of bias were minimized by including all eligible surgical patients during the study period and by controlling for confounders using multivariable logistic regression analysis.

### 2.12. Data Processing and Analysis

The data were checked for accuracy and exported from Kobo Toolbox. Statistical analyses were performed using IBM SPSS Statistics for Windows, Version 27 (IBM Corp., Armonk, New York, USA). Descriptive statistics (frequency, percentage, mean, and standard deviation) were used to summarize the variables. The findings were presented using tables, figures, and narrative descriptions. Model fitness was assessed using the Hosmer–Lemeshow goodness‐of‐fit test, with *p* > 0.05 indicating an adequate fit. Bivariate and multivariable logistic regression analyses were performed to identify factors associated with the risk of SSIs. Variables with *p* < 0.25 in the bivariate analysis were included in the multivariable model to control for potential confounders. The magnitude of association between independent variables and the outcome was expressed as adjusted odds ratios (AORs) with 95% confidence intervals (CIs), and statistical significance was defined as *p* < 0.05. All covariates tested in the bivariate analysis are reported in the result section.

### 2.13. Operational Definitions

SAP is defined as the administration of an antibiotic within 60 min prior to a surgical incision, and when indicated, for a very short course after surgery to prevent SSIs [24].

**SSI** is termed as an infection occurring at or near the surgical site within 30 days after the operation, or within 90 days for implant procedures that meets the CDC criteria for superficial incisional, deep incisional, or organ/space infections [7].

### 2.14. Reporting Guidelines

This study is reported in accordance with the Strengthening the Reporting of Observational Studies in Epidemiology (STROBE) guidelines, and the completed STROBE checklist is provided (Supporting File 1).

## 3. Results

### 3.1. Study Participants

**Figure 1:**
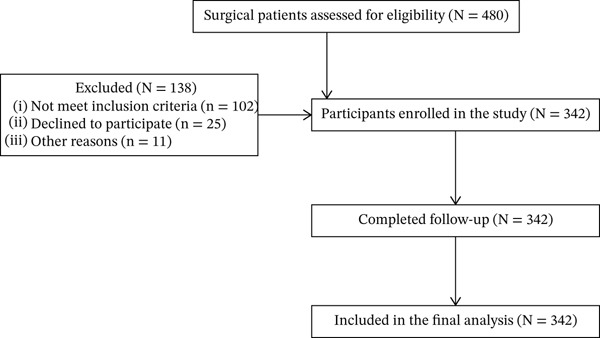
Flow diagram of surgical patients assessed for eligibility, enrolled, and included in the final analysis at DTCSH from February 1 to May 30, 2025 (*N* = 480). The figure depicts the participant flow diagram showing the number of surgical patients assessed for eligibility, excluded, enrolled, and included in the final analysis (*N* = 342). Note: Lost to follow‐up/withdrawn (*N* = 0).

Among a total of 480 surgical patients assessed for eligibility, 138 were excluded for not meeting inclusion criteria, declining participation, or other reasons. The remaining 342 participants were enrolled, all of whom completed follow‐up and were included in the final analysis (Figure 1).

### 3.2. Sociodemographic and Clinical Characteristics of Patients

Among 342 participants, more than half were female (55%). The mean age was 36.2 ± 13.5 years, ranging from 18 to 78. Most participants were from rural areas (62%), and nearly two‐thirds were married (63.7%). Regarding clinical characteristics, 190 procedures (55.6%) were elective, and 297 patients (86.8%) had no comorbidities. Operation durations were < 1 h in 31%, 1–2 h in 47.7%, 2–3 h in 17.5%, and > 3 h in 3.8% (Table 1).

**Table 1:** Sociodemographic and clinical characteristics of surgical patients at DTCSH from February 1 to May 30, 2025 (*N* = 342).

Variables	Category	Frequency (*n*)	Percent (%)	*M* *e* *a* *n* ± *S* *D*	Range
Age	—	—	—	36.23 ± 13.47	18–78
Gender	Male	154	45.0	—	—
	Female	188	55.0	—	—
Residence	Urban	130	38.0	—	—
	Rural	212	62.0	—	—
Marital status	Single	101	29.6	—	—
	Married	218	63.7	—	—
	Widowed	13	3.8	—	—
	Divorced	10	2.9	—	—

Type of admission	Emergency	152	44.4	—	—
	Elective	190	55.6	—	—
Comorbid diseases	Yes	45	13.2	—	—
	No	297	86.8	—	—
Duration of surgery	< 1 h	106	31.0	—	—
	1–2 h	163	47.7	—	—
	2–3 h	60	17.5	—	—
	> 3 h	13	3.8	—	—

### 3.3. Wound Classification

**Figure 2:**
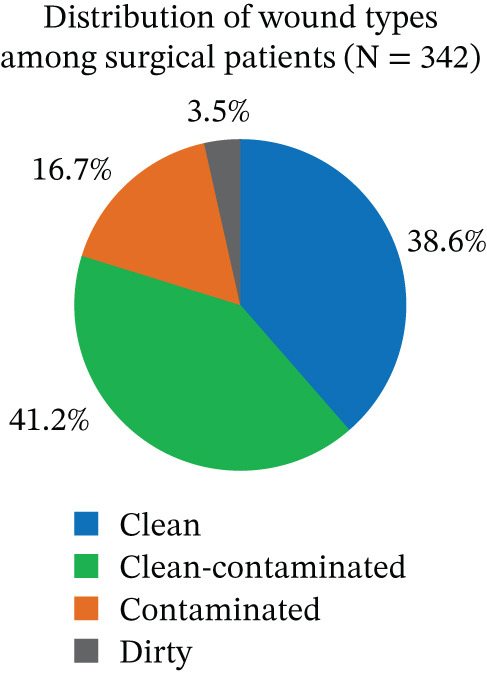
Percentage distribution of surgical cases by wound type among surgical patients at DTCSH from February 1 to May 30, 2025 (*N* = 342). The pie chart displays the proportion of surgical cases among the study subjects (*N* = 342) by wound classification (clean, clean‐contaminated, contaminated, and dirty).

Among the 342 respondents, clean‐contaminated wounds were the most common, observed in 141 cases (41.2%), followed by clean wounds in 132 patients (38.6%) (Figure 2).

### 3.4. Antibiotics Utilization in Surgical Wards

#### 3.4.1. Preoperative Antibiotic Prophylactic Use

**Figure 3:**
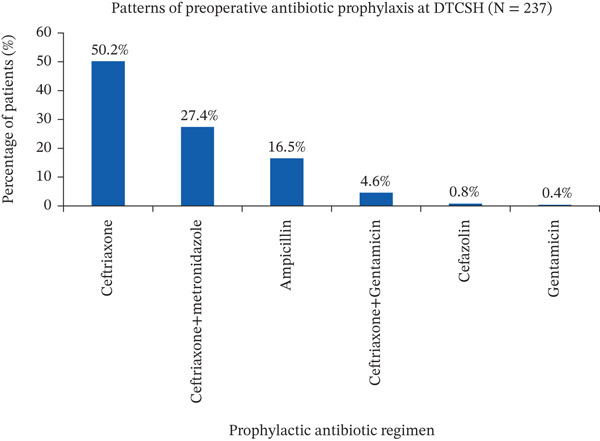
Distribution of preoperative prophylactic antibiotics among surgical patients at DTSCH from February 1 to May 30, 2025 (*N* = 237). The bar chart shows the percentage of cases receiving each antibiotic regimen (ceftriaxone, ceftriaxone plus metronidazole, ampicillin, ceftriaxone plus gentamicin, cefazolin, and gentamicin) among surgical patients (*N* = 237).

Of all patients admitted for surgical procedures, 237 (69.3%) received preoperative SAP (Table 2). Ceftriaxone was the most commonly used agent, administered to 119 patients (50.2%), followed by a combination of ceftriaxone and metronidazole in 65 patients (27.4%) (Figure 3). Among patients who received SAP, 161 (67.9%) were treated with a single agent, whereas 76 (32.1%) received a combination of two antibiotics. All preoperative antibiotics were administered intravenously (Table 3).

**Table 2:** SAP status of surgical patients at DCSH from February 1 to May 30, 2025 (*N* = 342).

Variable	Category	Frequency	Percent (%)
Antibiotic prophylaxis status	Yes	237	69.3
	No	105	30.7

**Table 3:** Number of prophylactic antibiotics administered and route of administration among surgical patients at DTCSH from February 1 to May 30, 2025 (*N* = 237).

Variable	Category	Frequency	Percent (%)
Number of antibiotics given	One	161	67.9
	Two	76	32.1
Route of administration	IV	237	100
	PO	0	0

#### 3.4.2. Timing of Antibiotic Prophylaxis Administration

**Figure 4:**
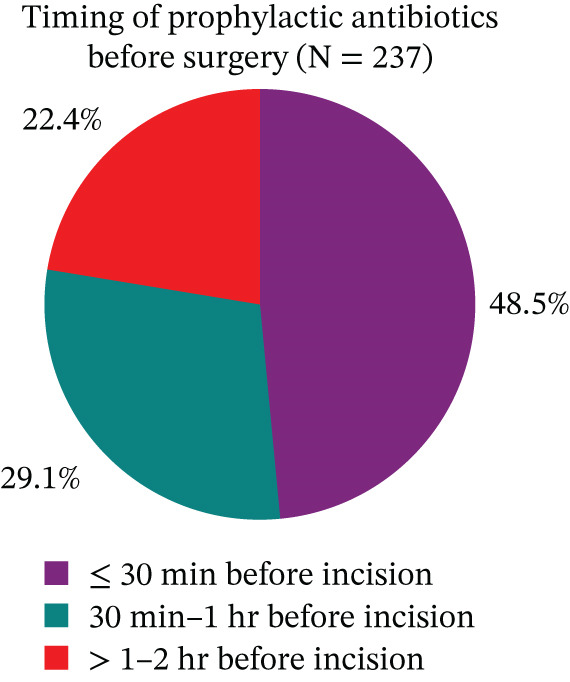
Timing of prophylactic antibiotic administration prior to surgical incision among surgical patients at DTCSH from February 1 to May 30, 2025 (*N* = 237). The pie chart illustrates the proportion of surgical cases among study subjects (*N* = 237) according to the timing of antibiotic administration relative to surgical incision: ≤ 30 min, 30 min–1 h, and > 1–2 h before incision.

Among patients who received prophylactic antibiotics, 115 (48.5%) received the drugs within 30 min before surgical incision, 69 (29.1%) between 30 min and 1 h prior to incision, and 53 (22.4%) between 1 and 2 h before incision (Figure 4).

#### 3.4.3. Postoperative Antibiotics Use

Of the 342 surgical patients, 122 (35.7%) received postoperative antibiotics. The most frequently prescribed antibiotics were the combination of ceftriaxone and metronidazole (57.4%), followed by ceftriaxone alone (8.2%) and ceftriaxone with gentamicin (6.6%). The majority of antibiotics were administered intravenously (86.1%) and primarily for prophylactic purposes (78.7%). Two‐drug combinations were the most frequently used regimen (79.5%) (Table 4).

**Table 4:** Postoperative antibiotic utilization among surgical patients at DTCSH from February 1 to May 30, 2025 (*N* = 342).

Variables	Category	Frequency	Percent (%)
Postoperative prophylaxis status	Yes	122	35.7
	No	220	64.3
Antibiotics given	Augmentin	4	3.3
	Ceftriaxone	10	8.2
	Ceftriaxone + Augmentin	3	2.5
	Ceftriaxone + Cloxacillin	5	4.1
	Ceftriaxone + Gentamicin	8	6.6
	Ceftriaxone + Gentamicin +	1	0.8
	Cloxacillin		
	Ceftriaxone + Metronidazole	70	57.4
	Ciprofloxacin	6	4.9
	Cloxacillin	6	4.9
	Gentamicin	5	4.1
	Vancomycin	4	3.3
Routes of administration	IV	105	86.1
	PO	11	9.0
	IV + PO	6	4.9
Purpose of antibiotics given	Treatment	26	21.3
	Prophylaxis	96	78.7
Basis of treatment	Empiric	26	100
Number of antibiotics used	One	24	19.7
	Two	97	79.5
	> Two	1	0.8

#### 3.4.4. Duration of Postoperative Antibiotic Prophylaxis

**Figure 5:**
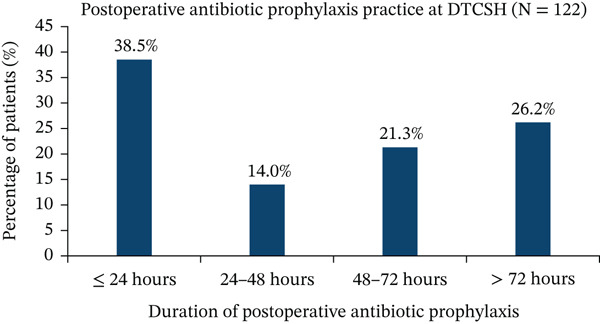
Distribution of postoperative antibiotic prophylaxis duration among surgical patients at DTCSH from February 1 to May 30, 2025 (*N* = 122). The bar chart depicts the percentage of patients receiving postoperative antibiotic prophylaxis for ≤ 24 h, 24–48 h, 48–72 h, and > 72 h among surgical patients (*N* = 122).

Among patients who received postoperative SAP, only 47 (38.5%) were administered antibiotics within the recommended 24‐h period. The majority (61.5%) received antibiotics for more than 24 h, with 17 patients (14.0%) receiving them for 24–48 h, 26 (21.3%) for 48–72 h, and 32 (26.2%) for more than 72 h (Figure 5).

#### 3.4.5. Evaluation of Antibiotic Prophylaxis Pattern

Out of 342 surgical patients, 237 (69.3%) had a clear indication for antibiotics and received them accordingly. Among patients without an indication, 99 (28.9%) did not receive antibiotics, whereas 6 (1.8%) were given antibiotics unnecessarily. The choice of SAP was inappropriate in 202 patients (83.1%). Among those who received antibiotics, the dosage was correct in 238 patients (97.9%) and the duration of prophylaxis was appropriate in 182 patients (74.9%). All SAP was administered via the recommended route (Table 5).

**Table 5:** Assessment of SAP practice among surgical patients at DTCSH from February 1 to May 30, 2025 (*N* = 243).

Variables	Category	Frequency	Percent (%)
Use of antibiotics	Indicated and administered	237	69.3
	Not indicated and administered	99	28.9
	Not indicated but administered	6	1.8
Antibiotic selection	Appropriate	41	16.9
	Inappropriate	202	83.1
Dosage appropriateness	Accurate	238	97.9
	Inaccurate	5	2.1
Duration of prophylaxis	Correct	182	74.9
	Incorrect	61	25.1
Route of administration	Correct	243	100

### 3.5. Development of SSI

Among the respondents, 37 (10.8%) developed postoperative SSIs; 13 (35.1%) were male and 24 (64.9%) were female. Most SSIs occurred following emergency procedures (26; 70.3%), with the remainder associated with elective surgeries. In terms of wound classification, clean‐contaminated wounds were the most common (13; 35.1%), followed by contaminated (10; 27.0%), clean (9; 24.3%), and dirty wounds (5; 13.5%).

### 3.6. Predictors Associated With SSIs

Multivariable logistic regression analysis was performed to identify independent predictors of SSIs. The Hosmer–Lemeshow goodness‐of‐fit test indicated good model fit (*χ* ^2^ = 6.42, *d* *f* = 8, *p* = 0.60), confirming that the model adequately described the observed data. As shown in Table 6, several factors were significantly associated with SSI. Elective surgery (*A* *O* *R* = 0.57, 95% CI: 0.19–0.86) and absence of comorbidities (*A* *O* *R* = 0.13, 95% CI: 0.09–0.75) were associated with a reduced risk of SSI. Longer surgical duration increased the risk, particularly for procedures lasting 2–3 h (*A* *O* *R* = 4.21, 95% CI: 3.47–13.84) and more than 3 h (*A* *O* *R* = 5.26, 95% CI: 4.52–15.91). Compared with clean wounds, contaminated (*A* *O* *R* = 3.42, 95% CI: 2.93–13.69) and dirty wounds (*A* *O* *R* = 9.35, 95% CI: 8.97–18.75) significantly increased risk. Proper use of prophylactic antibiotics reduced SSI risk (*A* *O* *R* = 0.67, 95% CI: 0.54–0.98), whereas administration more than 1–2 h before incision (*A* *O* *R* = 5.34, 95% CI: 5.01–16.12) or continuation beyond 72 h postoperatively (*A* *O* *R* = 5.63, 95% CI: 5.35–14.27) significantly increased the odds of SSI.

**Table 6:** Multivariable logistic regression analysis of factors associated with SSI among surgical patients at DTCSH from February 1 to May 30, 2025 (*N* = 342).

Variables	Categories	SSI status		Bivariate analysis	Multivariate analysis
		Yes (%)	No (%)	COR (95% CI)	AOR (95% CI)
Age	—	—	—	1.03 (0. 79, 1.07) ^∗∗^	1.32 (0.84, 1.69)
Sex	Male	17 (11.1)	137 (88.9)	1	—
	Female	20 (10.6)	168 (89.4)	0.96 (0.48, 1.90)	—
Residence	Urban	10 (7.6)	120 (92.4)	1	1
	Rural	27 (12.7)	185 (87.3)	1.75 (0.82, 3.82) ^∗∗^	2.13 (0.98, 3.57)
Type of surgery	Emergency	26 (17.1)	126 (82.9)	1	1
	Elective	11 (5.8)	179 (94.2)	0.29 (0.14, 0.63) ^∗∗^	0.57 (0.19, 0.86) ^∗^
Comorbidity status	Yes	13 (28.9)	32 (71.1)	1	1
	No	24 (8.1)	273 (91.9)	0.21 (0.10, 0.47) ^∗∗^	0.13 (0.09, 0.75) ^∗^
Duration of surgery	< 1 h	7 (6.6)	99 (93.4)	1	1
	1–2 h	13 (8.1)	150 (91.9)	1.23 (0.47, 3.16)	1.34 (0.97, 1.46)
	2–3 h	13 (21.7)	47 (78.3)	3.91 (1.47, 10.45) ^∗∗^	4.21 (3.47, 13.84) ^∗^
	> 3 h	4 (30.8)	9 (69.2)	6.29 (1.54, 25.6) ^∗∗^	5.26 (4.52, 15.91) ^∗^
Wound class	Clean	9 (6.8)	123 (93.2)	1	1
	Clean contaminated	13 (9.2)	128 (90.8)	1.37 (0.57, 3.37) ^∗∗^	1.29 (0.85, 1.67)
	Contaminated	10 (17.6)	47 (82.4)	2.94 (1.11, 7.60) ^∗∗^	3.42 (2.93, 13.69) ^∗^
	Dirty	5 (41.7)	7 (58.3)	9.86 (2.57, 37.0) ^∗∗^	9.35 (8.97, 18.75) ^∗^
Prophylactic antibiotic	Inappropriate	35 (17.3)	167 (82.7)	1	1
	Appropriate	2 (4.9)	39 (95.1)	0.24 (0.06, 0.98) ^∗∗^	0.67 (0.54, 0.98) ^∗^
Dosing time of SAP	≤ 30 min	11 (9.6)	104 (90.4)	1	1
	30 min–1 h	9 (13.1)	60 (86.9)	1.49 (0.56, 3.62)	1.20 (0.89, 1.91)
	> 1–2 h	17 (32.1)	36 (67.9)	4.47 (1.91, 10.42) ^∗∗^	5.34 (5.01, 16.12) ^∗^
Duration of SAP	≤ 24 h	9 (19.1)	38 (80.9)	1	1
	> 24–48 h	4 (23.5)	13 (76.5)	1.30 (0.34, 4.93)	1.68 (0.93, 2.87)
	> 48–72 h	7 (26.9)	19 (73.1)	1.52 (1.02, 4.84) ^∗∗^	1.79 (0.78, 1.94)
	> 72 h	17 (53.1)	15 (46.9)	4.76 (1.75, 13.01) ^∗∗^	5.63 (5.35, 14.27) ^∗^

*Note:* 1 = Reference category.

Abbreviations: AOR, adjusted odds ratio; CI, confidence interval; COR, crude odds ratio; h, hour; SAP, surgical antibiotic prophylaxis; SSI, surgical site infection.

^∗^significant association at *p* value < 0.05.

^∗∗^significant association at *p* value < 0.25.

## 4. Discussion

### 4.1. Comparison With Existing Studies

In the present study, most patients were admitted to the general surgery ward (59.4%), consistent with findings from India (62%) [25] and Nekemte Hospital, Ethiopia (60.1%) [26]. In contrast, higher proportions were observed in a multicenter survey from Ghana (71.5%) [27], whereas a lower proportion was reported at Saint Paul Hospital, Addis Ababa (36.8%) [28], likely reflecting differences in hospital structure and the availability of specialized surgical wards.

Regarding surgical prophylaxis, 69.3% of patients received preoperative antibiotics, similar to reports from the Netherlands (70%) [29], India (67%) [25], and Black Lion Specialized Hospital, Ethiopia (68.7%) [30]. Nonetheless, a higher prevalence was documented at Hiwot Fana Comprehensive Specialized Hospital, Ethiopia (86.2%) [31], suggesting institutional differences in prophylaxis protocols and adherence to standard guidelines. Ceftriaxone was the most commonly used preoperative antibiotic (50.2%), followed by ceftriaxone plus metronidazole (27.4%). This differs from findings in Northwest Iran, where cefazolin was utilized in 90% of cases [32]. However, comparable prescribing patterns have been reported at Dessie Referral Hospital, Northeast Ethiopia, where 53.6% of patients received ceftriaxone [20], indicating consistency with local practice.

Among patients receiving postoperative antibiotics, 78.3% were prescribed for prophylaxis and 21.7% for treatment. Globally, high rates of postoperative prophylaxis have been reported, including 92% in Italy [33], 84.2% in Bangladesh [34], and 77.5% in India [35]. Similar patterns have been observed in Tanzania, where 79.2% of patients received postoperative antibiotics, predominantly ceftriaxone alone (85.7%) [36]. In this study, ceftriaxone plus metronidazole was the predominant postoperative regimen (69.6%), in line with findings from Saint Paul Hospital, Addis Ababa (72%) [28]. These findings suggest that ceftriaxone‐based regimens remain widely used despite guideline recommendations favoring first‐generation cephalosporins.

Evaluation of SAP practices showed that 69.3% of patients had an appropriate indication and received prophylactic antibiotics. By comparison, 28.9% had no indication and appropriately did not receive prophylaxis, whereas 1.8% received prophylactic antibiotics despite the absence of an indication. Previous studies have reported wide variability in inappropriate prophylactic antibiotic use, with rates as high as 44% in Iran and 35.7% in Italy [37, 38]. Whereas at Black Lion Specialized Hospital, Ethiopia, 87.4% appropriate use and 12.6% unnecessary administration were reported [39]. These findings indicate that inappropriate prophylactic antibiotic use persists across healthcare settings. Ceftriaxone was the most frequently used agent for surgical prophylaxis (50.2%), despite guideline recommendations favoring cefazolin as the first‐line option for most surgical procedures. These findings highlight the urgent need for antimicrobial stewardship interventions, including the strengthened guideline implementation and targeted prescriber education, to optimize surgical outcomes.

Improper use of prophylactic antibiotics increases the risk of antimicrobial resistance, adverse drug reactions, unnecessary healthcare costs, and SSIs [40]. In our cohort, only 16.9% of patients received appropriate antibiotics for surgical prophylaxis. Globally, the appropriateness of SAP varies widely, with appropriate use documented in 70.3%–95% of procedures and inappropriate use ranging from 2.3% to 100% [41]. Compared with the 22.3% rate observed at Tibebe Ghion Specialized Hospital, Ethiopia [42], our findings demonstrate poor compliance with recommended surgical prophylaxis practices.

Timely administration of prophylactic antibiotics is essential for SSI prevention, ideally within 1 h before incision or up to 2 h for agents requiring longer infusion [43]. In our study, 77.6% of patients received SAP within 1 h prior to incision. Relative to this, a review of 14 global studies showed adherence rates ranging from 12.7% to 100% [41], with Dutch hospitals showing 51% [29] and 90.2% adherence observed at Saint Paul Hospital, Ethiopia [28].

The recommended duration of SAP is generally within 24 h, with a single preincision dose often sufficient [44]. In this analysis, 38.5% of patients received prophylaxis for less than 24 h, compared with 56% documented in Bangalore, India [45], suggesting an inadequate adherence to standard guidelines. Regarding dosing, 97.9% of patients received the correct dose, similar to compliance observed at Razi Hospital, Iran (91.8%) and Saint Paul Hospital, Ethiopia (100%) [28, 37]. Additionally, all patients received SAP via the recommended route, aligning with findings from India [46], Pakistan [47], and Ethiopia [23], reflecting a strong adherence to ASHP guidelines.

SSI risk is influenced by surgery type, patient health, and quality of care, and it is a recognized global health problem [48, 49]. According to multiple studies, prevalence varies widely, ranging from 2.5% to 41.9% [50]. In our study, SSI occurred in 10.8% of patients, comparable with 9.02% at Zewditu Memorial Hospital [51] but lower than rates reported at Finote Selam General Hospital (19.6%), Felege Hiwot Referral Hospital (14.4%), and in Kenya (22%) [52–54]. These variations could likely reveal differences in surgical case mix, wound contamination profiles, and patient comorbidities.

### 4.2. Possible Biological or Operational Explanations

The study was conducted at a single public hospital for 4 months using consecutive sampling. This approach was chosen due to resource constraints, the feasibility of close supervision, the availability of trained data collectors, and the high surgical load during the study period. Conducting the study at a single site also ensured consistency in clinical practice, simplified ethical approvals, and allowed timely completion of data collection, while including all eligible patients minimized selection bias.

In the study setting, ceftriaxone alone or in combination with metronidazole was the most frequently used antibiotic for surgical prophylaxis instead of the guideline‐recommended cefazolin. Limited availability of cefazolin, lack of locally adapted SAP guidelines, habitual broad‐spectrum prescribing, prescriber knowledge gaps, and institutional norms may likely contribute to this practice.

Our analysis identified several factors independently associated with SSIs. Elective surgeries were associated with a 43% lower risk compared with emergency procedures, likely due to more timely SAP administration and a lower likelihood of involving contaminated or dirty wounds [55]. Patients without comorbidities were 87% less likely to develop SSIs than those with comorbidities. This may be explained by intact immune function, better tissue perfusion, and faster wound healing. In addition, noncomorbid individuals are less likely to require prolonged procedures, invasive devices, or immunosuppressive medications, all of which can increase the risk of infection in patients with comorbidities.

Prolonged surgical duration was associated with a significantly higher risk of SSIs, with procedures lasting 2–3 h and over 3 h showing 4.2‐ and 5.3‐fold increased risk, respectively. This may be attributed to greater tissue trauma and microbial exposure, consistent with global evidence [56] and several Ethiopian studies [20, 57, 58]. Similarly, contaminated and dirty wounds had 3.4‐ and 9.3‐fold higher risk compared with clean wounds, aligned with a study linking wound classification to SSIs [59]. These findings are likely due to high microbial load, tissue devitalization, impaired host defenses, and reduced efficacy of prophylactic antibiotics.

SAP practices were also associated with SSI development. Appropriate SAP reduced SSI risk by 33%, in concordance with studies from Egypt and Ethiopia [40, 57, 60]. Conversely, patients receiving SAP more than 1–2 h before incision had a 5.3‐fold higher risk than those who received it within 30 min, aligning with global and Ethiopian studies reporting increased SSI risk when antibiotics are administered too early [52, 61, 62]. This likely reflects suboptimal antibiotic concentrations at the time of incision, thereby reducing prophylactic effectiveness and increasing susceptibility to bacterial contamination. Studies confirmed that administering SAP too early leads to low antibiotic levels at the time of incision [63]. Similarly, prolonged postoperative SAP (> 72 h) was associated with a 5.6‐fold increase in SSI risk, potentially due to the emergence of resistant organisms and subsequent secondary infections [64].

### 4.3. Implications for Practice and Policy

This study highlights significant gaps between current SAP practices at DTCSH and guideline recommendations. To reduce SSIs, surgical teams should adhere to recommended antibiotic selection, timing, and duration, while hospitals should ensure consistent access to essential drugs such as cefazolin and actively involve clinical pharmacists in perioperative care. Key patient and procedure‐related factors, including comorbidities, wound type, and duration of surgery, should be considered during surgical planning. Health facilities should strengthen perioperative documentation, implement antimicrobial stewardship programs, and routinely monitor SSI rates. Policymakers should promote adoption of standardized SAP protocols, establish monitoring systems, and support ongoing training of surgical teams in SAP best practices and SSI prevention.

### 4.4. Limitations and Future Directions for Research

This study has certain limitations. Data were collected from a single hospital over a 4‐month period using consecutive sampling, which may limit generalizability. Important variables, including surgeon experience, preoperative hospital stay, wound drainage, and adherence to aseptic technique, were not included in the analysis, potentially introducing residual confounding. Future studies should adopt multicenter designs, longer follow‐up periods, probability‐based sampling, and broader variable assessment to strengthen the robustness of the findings.

## 5. Conclusions

A significant discrepancy exists between SAP practices at DTCSH and established guidelines, with inappropriate antibiotic selection being the most common deviation. The relatively high SSI burden and the identification of multiple independent predictors, including type of surgery, comorbidities, procedure duration, wound type, and SAP selection, timing, and duration, underscore the urgent need to optimize perioperative care. Surgical teams should adhere strictly to evidence‐based guidelines, actively involve clinical pharmacists, and ensure consistent availability of essential antibiotics such as cefazolin. Future multicenter studies are warranted to enhance generalizability and further support guideline‐based surgical prophylaxis.

**Nomenclature**
AMR: antimicrobial resistance
AOR: adjusted odds ratio
ASHP: American Society of Health System Pharmacists
CDC: centers for disease control
CI: confidence interval
DTCSH: Debre Tabor Comprehensive Specialized Hospital
SAP: surgical antibiotic prophylaxis
SPSS: Statistical Package for the Social Sciences
SSIs: surgical site infections
WHO: World Health Organization

## Author Contributions

All authors contributed significantly to this work. Y.A.F.: conceptualization, data collection, and writing—review & editing; M.A.A.: formal analysis, data curation, and supervision; S.B.D.: conceptualization and methodology; T.A.M.: validation and writing—original draft; A.B.K.: data collection and writing—review & editing; W.S.Z.: methodology, supervision, and formal analysis; A.B.M.: conceptualization and validation.

## Funding

No funding was received for this manuscript.

## Disclosure

All authors agree to take full responsibility for the content and findings of this study.

## Ethics Statement

The study was approved by the Ethical Review Committee of the College of Health Sciences, Debre Tabor University (Reference No: CHS/1/107/2025) and an official permission letter was granted from the medical director of DTCSH. Written informed consent was received from each participant and/or their legal guardians before data collection, and consent was documented using a standardized form. Patient confidentiality was maintained throughout the study by omitting names and other personal identifiers. Data extracted from medical charts were used solely for research purposes.

## Conflicts of Interest

The authors declare no conflicts of interest.

## Supporting information


**Supporting Information** Additional supporting information can be found online in the Supporting Information section. This file contains the completed Strengthening the Reporting of Observational Studies in Epidemiology (STROBE) checklist, indicating the specific page numbers at which each reporting item is addressed in the manuscript.

## Data Availability

The data analyzed or generated in this study can be accessed by contacting the corresponding author, subject to a reasonable request
