# Supplementary material for: Surgical Antibiotic Prophylaxis Utilization and Predictors of Surgical Site Infections at Debre Tabor Comprehensive Specialized Hospital, Northwest Ethiopia: Prospective Cohort Study
Source: Biomed Res Int. 2026 Feb 24;2026:6612112. doi: 10.1155/bmri/6612112 (PMC12932327; doi:10.1155/bmri/6612112)
Supplement: Supplementary file 1 — Supporting Information Additional supporting information can be found online in the Supporting Information section. This file contains the completed Strengthening the Reporting of Observational Studies in Epidemiology (STROBE) checklist, indicating the specific page numbers at which each reporting item is addressed in the manuscript. [file BMRI-2026-6612112-s001.docx]

STROBE Statement—Checklist of items that should be included in reports of **observational *studies***

|  | Item No | Recommendation | Page No |
| --- | --- | --- | --- |
| **Title and abstract** | 1 | (*a*) Indicate the study’s design with a commonly used term in the title or the abstract | Surgical Antibiotic Prophylaxis Utilization and Predictors of Surgical Site Infections at Debre Tabor Comprehensive Specialized Hospital, Northwest Ethiopia: Prospective Cohort Study (**Title section, page 1, line no 3**). A prospective cohort study was conducted among 342 surgical patients from February 1 to May 30, 2025 (**abstract section, page no 2, line no 29).** |
|  |  | (*b*) Provide in the abstract an informative and balanced summary of what was done and what was found | A prospective cohort study was conducted among 342 surgical patients from February 1 to May 30, 2025. Adherence of surgical antibiotic prophylaxis was assessed based on standard national and international guidelines. Descriptive, bivariate, and multivariable analyses were conducted using IBM SPSS software version 27. Statistical significance was set at p <0.05 (**Method section, page no 2, line no 29 up to 32**). Preoperative prophylaxis was administered to 69.3% of patients, although 83.1% received inappropriate antibiotics. Ceftriaxone (50.2%) and its combination with metronidazole (27.4%) were the most commonly used antibiotics. The overall magnitude of SSI was 10.8% (95% CI: 7.5–14.1). Elective surgery (AOR = 0.57), absence of comorbidities (AOR = 0.13), and appropriate SAP use (AOR = 0.67) were associated with reduced odds of developing SSI. In contrast, procedures lasting 2–3 hours (AOR = 4.21) or more than 3 hours (AOR = 5.26), contaminated (AOR = 3.42) or dirty wounds (AOR = 9.35), antibiotic prophylaxis administered more than 1–2 hours before incision (AOR = 5.34), and postoperative SAP duration exceeding 72 hours (AOR = 5.63) were linked to an increased risk of SSIs (**Result section, page no 2, line no 33 up to 41**). |
| Introduction | | | |
| Background/rationale | 2 | Explain the scientific background and rationale for the investigation being reported | **Background**: SSIs are defined as infections occurring at or near the surgical incision within 30 days postoperatively, or within 90 days if an implant is placed (7). They are among the most common healthcare-associated infections in surgical patients, ranking second among hospital-acquired infections. SSIs account for approximately 14–17% of all hospital-acquired infections and 38% of infections in surgical patients (8,9). In low- and middle-income countries (LMICs), SSI rates range from 8% to 30% of surgical procedures, representing the most frequent hospital-acquired infection and contributing substantially to morbidity, mortality, and economic burden (10). SAP plays a critical role in preventing SSIs, which are among the most common postoperative complications (14). However, improper use such as unnecessary administration, incorrect antibiotic selection, inappropriate timing, and prolonged duration remains a significant concern (15). Studies indicate that 30–90% of SAP prescriptions are inappropriate (16). Postoperative wound infections occur in approximately 15% of elective surgeries and up to 30% of contaminated or dirty procedures (17). Globally, the pooled incidence of SSIs is reported to be 2.5% (18), whereas a meta-analysis in sub-Saharan Africa reported higher rates, ranging from 6.8% to 26% (19) (**Introduction section, page no 3 & 4, line no 58 up to 78**). **Rationale**: Despite global and national efforts to reduce SSIs through evidence-based guidelines, including appropriate SAP, significant challenges persist in resource-limited settings such as Ethiopia (20). Few studies have addressed this issue in these contexts, and none have been conducted in the current study setting. Therefore, this study aimed to assess the patterns of prophylactic antibiotic use, the magnitude of SSIs, and their associated predictors in the surgical ward of Debre Tabor Comprehensive Specialized Hospital (DTCSH). The findings are intended to identify gaps in SAP practices, provide local evidence to guide hospital policies, and serve as a baseline for future interventions, ultimately contributing to improved SSI control and the appropriate use of SAP (**Introduction section, page no 4, line no 79 up to 87**). |
| Objectives | 3 | State specific objectives, including any prespecified hypotheses | This study aimed to assess the patterns of prophylactic antibiotic use, the magnitude of SSIs, and their associated predictors in the surgical ward of Debre Tabor Comprehensive Specialized Hospital (DTCSH) (**Introduction section, page no 4, line no 82 up to 84**). |
| Methods | | | |
| Study design | 4 | Present key elements of study design early in the paper | A prospective cohort study was conducted among patients undergoing surgery at DTCSH from February 1 to May 30, 2025. (**Method section, page no 4, line no 96 & 97**). |
| Setting | 5 | Describe the setting, locations, and relevant dates, including periods of recruitment, exposure, follow-up, and data collection | The study was conducted in the surgical wards of DTCSH, located in Debre Tabor Town, South Gondar Zone, Amhara Region, Northwest Ethiopia (**method section, page 4, line no 90 & 91**). Eligible patients were enrolled at the time of their surgical procedure and followed for 30 days, or up to 90 days for implant procedures, to determine the occurrence of SSIs (**Method section, page 4, line no 97 up to 99**). |
| Participants | 6 | (*a*) Give the eligibility criteria, and the sources and methods of selection of participants | **2.5. Inclusion criteria**  All adult patients (≥18 years) admitted to DTCSH for major surgical procedures during the study period with a postoperative hospital stay of at least 48 hours were eligible for inclusion (**method section, page no 5, line no 109 & 110**).  **2.6. Exclusion criteria**  Patients who underwent minor surgical procedures, were operated on elsewhere and later referred to DTCSH, had preexisting SSIs, or had missing essential information were excluded from the study (**Method section, page no 5, line no 112 up to 114**). Consecutive sampling was employed, including all eligible surgical patients admitted during the study period until the required sample size was achieved (**method section, page 5, line no 128 & 129**). |
| Variables | 7 | Clearly define all outcomes, exposures, predictors, potential confounders, and effect modifiers. Give diagnostic criteria, if applicable | The primary outcome variable in this study was the occurrence of SSI. Independent variables included sociodemographic characteristics (age, sex, and marital status), clinical factors (presence of comorbidities, duration of operation, type of admission, and wound classification), and antibiotic-related variables (use and choice of prophylactic antibiotics, timing of administration, dosage regimen, and postoperative antibiotic use duration). (**Method section, page no 6, line no 131 up to 135**). |
| Data sources/ measurement | 8* | For each variable of interest, give sources of data and details of methods of assessment (measurement). Describe comparability of assessment methods if there is more than one group | Sociodemographic data were collected through direct patient interviews, while clinical and other relevant information were extracted from medical charts. Postoperative wound assessments were conducted daily during hospitalization using a structured checklist adapted from the CDC SSI surveillance tool. These assessments were performed by trained operating room nurses, and any suspected SSI was confirmed by the attending surgeon. Assessment criteria included clinical signs such as purulent discharge, localized pain, redness, swelling, increased warmth, and, when available, microbiological evidence.  After discharge, patients were followed for 30 days, and up to 90 days for implant-related procedures, in accordance with CDC recommendations. Post-discharge follow-up was conducted through scheduled outpatient clinic visits and telephone interviews for patients unable to return. All data were collected by two trained nurses under the close supervision of the principal investigator (**Method section, page no 6, line no 144 up to 155**). |
| Bias | 9 | Describe any efforts to address potential sources of bias | Before data collection, the tool was reviewed by three academic experts in pharmacy, public health, and infectious diseases to ensure content validity. A pretest was conducted at Woldia Comprehensive Specialized Hospital using 5% of the study population to identify ambiguous or unclear items, which were subsequently rephrased for clarity. Participants included in the pretest were excluded from the main study, and their data were not included in the final analysis to avoid contamination. Data collectors were trained on standardized procedures, and data were reviewed daily by the principal investigator for completeness and consistency. Records with missing key outcome variables were excluded from the analysis. Potential sources of bias were minimized by including all eligible surgical patients during the study period and by controlling for confounders using multivariable logistic regression analysis (**Method section, page no 7, line no 164 up to 173**) |
| Study size | 10 | Explain how the study size was arrived at | The sample size was determined using the single population proportion formula with a 95% confidence level (Z = 1.96), a margin of error of 5% (E), and an estimated population proportion (P) of 0.5:  n = $\frac{Z^{2}P(1-P)}{E^{2}}$  Accordingly, the initial sample size was calculated as follows:  n = (1.96) ^2^ × 0.5 × (1 − 0.5) = 384.16 ≈ 384  (0.05)^2^  Since the total number of patients admitted to the surgical wards over the preceding four months was fewer than 10,000 (N = 1,640), a finite population correction formula was applied:  nf = n / (1 + n/N) = 384 / (1+384/1640) = 311  Where nf = adjusted sample size, n = initial sample size, and N = total population size.  To account for potential incomplete data and response errors, 10% was added, resulting in a final sample size of 342 participants (**method section, page no 5, line no 116 up to 128**). |
| Quantitative variables | 11 | Explain how quantitative variables were handled in the analyses. If applicable, describe which groupings were chosen and why | Among the study variables, age, duration of surgery, timing of SAP administration, and duration of post-operative prophylaxis were quantitative variables. From these, age was treated as a continuous variable and entered into the regression model as years. While, the rest variables were categorized based on the previous studies (**Method section, page no 6, line no 130 up to 135**). |
|  | 12 | (*a*) Describe all statistical methods, including those used to control for confounding | The data were checked for accuracy and exported from Kobo Toolbox. Statistical analyses were performed using IBM SPSS Statistics for Windows, version 27 (IBM Corp., Armonk, NY, USA). Descriptive statistics (frequency, percentage, mean, and standard deviation) were used to summarize the variables. The findings were presented using tables, figures, and narrative descriptions. Model fitness was assessed using the Hosmer–Lemeshow goodness-of-fit test, with p > 0.05 indicating an adequate fit. Bivariate and multivariable logistic regression analyses were performed to identify factors associated with the risk of SSIs. Variables with p < 0.25 in the bivariate analysis were included in the multivariable model to control for potential confounders. The magnitude of association between independent variables and the outcome were expressed as adjusted odds ratios (AORs) with 95% confidence intervals (CIs), and statistical significance was defined as p < 0.05 (**Method section, page no 7, line no 175 up to 185**). |
|  |  | (*b*) Describe any methods used to examine subgroups and interactions | No formal interaction and subgroup analyses were conducted. |
|  |  | (*c*) Explain how missing data were addressed | The study was conducted prospectively, and all participants were followed from enrollment to study completion. All study variables were recorded in real time using standardized data collection forms. Immediate verification and monitoring of the forms ensured that no data were missing for any participant. Therefore, no imputation or other methods for handling missing data were required (**Methods section, page no 7, line no 158 up to 162**) |
|  |  | (*d*) If applicable, describe analytical methods taking account of sampling strategy | Not applicable |
|  |  | (*e*) Describe any sensitivity analyses | Not applicable: no sensitivity analyses was conducted |
| Results | | | |
| Participants | 13* | (a) Report numbers of individuals at each stage of study—eg numbers potentially eligible, examined for eligibility, confirmed eligible, included in the study, completing follow-up, and analysed | Among a total of 480 surgical patients assessed for eligibility, 138 were excluded for not meeting inclusion criteria, declining participation, or other reasons. The remaining 342 participants were enrolled, all of whom completed follow-up and were included in the final analysis (Figure 1). (**Result section, page no 8, line no 195 up to 197**). |
|  |  | (b) Give reasons for non-participation at each stage | Surgical patients were declined to participate due not meeting inclusion criteria, declining participation, or other reasons (**Result section, page no 8, line no 195 & 196**). |
|  |  | (c) Consider use of a flow diagram | Clearly shown in figure 1 (**method section, page no 8, line no 198 up to 208**) |
| Descriptive data | 14* | (a) Give characteristics of study participants (eg demographic, clinical, social) and information on exposures and potential confounders | Among 342 participants, more than half were female (55%). The mean age was 36.2 ± 13.5 years, ranging from 18 to 78. Most participants were from rural areas (62%), and nearly two-thirds were married (63.7%). Regarding clinical characteristics, 190 procedures (55.6%) were elective, and 297 patients (86.8%) had no comorbidities. Operation durations were <1 hour in 31%, 1–2 hours in 47.7%, 2–3 hours in 17.5%, and >3 hours in 3.8% (Table 1) (**Result section, page no 8, line no 215 up to 219**). |
|  |  | (b) Indicate number of participants with missing data for each variable of interest | All variables of interest were complete, and no missing data occurred. |
| Outcome data | 15* | Report numbers of outcome events or summary measures | Among the respondents, 37 (10.8%) developed postoperative SSIs; 13 (35.1%) were male and 24 (64.9%) were female. Most SSIs occurred following emergency procedures (26; 70.3%), with the remainder associated with elective surgeries. In terms of wound classification, clean-contaminated wounds were most common (13; 35.1%), followed by contaminated (10; 27.0%), clean (9; 24.3%), and dirty wounds (5; 13.5%) (**Result section, page no 15, line no 287 up to 291**). |
| Main results | 16 | (*a*) Give unadjusted estimates and, if applicable, confounder-adjusted estimates and their precision (eg, 95% confidence interval). Make clear which confounders were adjusted for and why they were included | As shown in Table 6, several factors were significantly associated with SSI. Elective surgery (AOR = 0.57, 95% CI: 0.19–0.86) and absence of comorbidities (AOR = 0.13, 95% CI: 0.09–0.75) were associated with a reduced risk of SSI. Longer surgical duration increased the risk, particularly for procedures lasting 2–3 hours (AOR = 4.21, 95% CI: 3.47–13.84) and more than 3 hours (AOR = 5.26, 95% CI: 4.52–15.91). Compared with clean wounds, contaminated (AOR = 3.42, 95% CI: 2.93–13.69) and dirty wounds (AOR = 9.35, 95% CI: 8.97–18.75) significantly increased risk. Proper use of prophylactic antibiotics reduced SSI risk (AOR = 0.67, 95% CI: 0.54–0.98), whereas administration more than 1–2 hours before incision (AOR = 5.34, 95% CI: 5.01–16.12) or continuation beyond 72 hours postoperatively (AOR = 5.63, 95% CI: 5.35–14.27) significantly increased the odds of SSI (**Result section, page no 15 & 16, line no 295 up to 305**). |
|  |  | (*b*) Report category boundaries when continuous variables were categorized | Age, duration of surgery, timing of SAP administration, and duration of SAP prophylaxis were continuous variables of all the independent variables, From this, the last 3 variables were categorized by referring the previous literature (Table 6) (**Result section, page no 16**). |
|  |  | (*c*) If relevant, consider translating estimates of relative risk into absolute risk for a meaningful time period | Not applicable since relative measures were the primary analytic objective due to complex multi variable modelling and Follow-up time is not uniform across study subjects. |
| Other analyses | 17 | Report other analyses done—eg analyses of subgroups and interactions, and sensitivity analyses | Not applicable. |
| Discussion | | | |
| Key results | 18 | Summarise key results with reference to study objectives | Regarding surgical prophylaxis, 69.3% of patients received preoperative antibiotics, similar to reports from the Netherlands (70%) (29), India (67%) (25), and Black Lion Specialized Hospital, Ethiopia (68.7%) (30). Nonetheless, a higher prevalence was documented at Hiwot Fana Comprehensive Specialized Hospital, Ethiopia (86.2%) (31), suggesting institutional differences in prophylaxis protocols and adherence to standard guidelines. Ceftriaxone was the most commonly used preoperative antibiotic (50.2%), followed by ceftriaxone plus metronidazole (27.4%). This differs from findings in Northwest Iran, where cefazolin was utilized in 90% of cases (32). However, comparable prescribing patterns have been reported at Dessie Referral Hospital, Northeast Ethiopia, where 53.6% of patients received ceftriaxone (20), indicating consistency with local practice.  Among patients receiving postoperative antibiotics, 78.3% were prescribed for prophylaxis and 21.7% for treatment. Globally, high rates of postoperative prophylaxis have been reported, including 92% in Italy (33), 84.2% in Bangladesh (34), and 77.5% in India (35). Similar patterns have been observed in Tanzania, where 79.2% of patients received postoperative antibiotics, predominantly ceftriaxone alone (85.7%) (36). In this study, ceftriaxone plus metronidazole was the predominant postoperative regimen (69.6%), in line with findings from Saint Paul Hospital, Addis Ababa (72%) (28). These findings suggest that ceftriaxone-based regimens remain widely used despite guideline recommendations favoring first-generation cephalosporins.  Evaluation of SAP practices showed that 69.3% of patients had an appropriate indication and received prophylactic antibiotics. By comparison, 28.9% had no indication and appropriately did not receive prophylaxis, while 1.8% received prophylactic antibiotics despite the absence of an indication. Previous studies have reported wide variability in inappropriate prophylactic antibiotic use, with rates as high as 44% in Iran and 35.7% in Italy (37,38). Whereas, at Black Lion Specialized Hospital, Ethiopia, 87.4% appropriate use and 12.6% unnecessary administration were reported (39). These findings indicate that inappropriate prophylactic antibiotic use persists across healthcare settings. Ceftriaxone was the most frequently used agent for surgical prophylaxis (50.2%), despite guideline recommendations favoring cefazolin as the first-line option for most surgical procedures. These findings highlight the urgent need for antimicrobial stewardship interventions, including strengthened guideline implementation and targeted prescriber education, to optimize surgical outcomes.  Improper use of prophylactic antibiotics increases the risk of antimicrobial resistance, adverse drug reactions, unnecessary healthcare costs, and SSIs (40). In our cohort, only 16.9% of patients received appropriate antibiotics for surgical prophylaxis. Globally, the appropriateness of SAP varies widely, with appropriate use documented in 70.3%–95% of procedures and inappropriate use ranging from 2.3% to 100% (41). Compared with the 22.3% rate observed at Tibebe Ghion Specialized Hospital, Ethiopia (42), our findings demonstrate poor compliance with recommended surgical prophylaxis practices.  Timely administration of prophylactic antibiotics is essential for SSI prevention, ideally within one hour before incision or up to two hours for agents requiring longer infusion (43). In our study, 77.6% of patients received SAP within one hour prior to incision. Relative to this, a review of 14 global studies showed adherence rates ranging from 12.7% to 100% (41), with Dutch hospitals showing 51% (29) and 90.2% adherence observed at Saint Paul Hospital, Ethiopia (28).  The recommended duration of SAP is generally within 24 hours, with a single pre-incision dose often sufficient (44). In this analysis, 38.5% of patients received prophylaxis for less than 24 hours, compared with 56% documented in Bangalore, India (45), suggesting inadequate adherence to standard guidelines. Regarding dosing, 97.9% of patients received the correct dose, similar to compliance observed at Razi Hospital, Iran (91.8%) , and Saint Paul Hospital, Ethiopia (100%) (28,37). Additionally, all patients received SAP via the recommended route, aligning with findings from India (46), Pakistan (47), and Ethiopia (23), reflecting strong adherence to ASHP guidelines.  SSI risk is influenced by surgery type, patient health, and quality of care, and it is a recognized global health problem (48,49). According to multiple studies, prevalence varies widely, ranging from 2.5% to 41.9% (50). In our study, SSI occurred in 10.8% of patients, comparable to 9.02% at Zewditu Memorial Hospital (51) but lower than rates reported at Finote Selam General Hospital (19.6%), Felege Hiwot Referral Hospital (14.4%), and in Kenya (22%) (52–54). These variations could likely reveal differences in surgical case mix, wound contamination profiles, and patient comorbidities (**Discussion section, page no 17, 18, and 19, line no 318 up to 376**). |
| Limitations | 19 | Discuss limitations of the study, taking into account sources of potential bias or imprecision. Discuss both direction and magnitude of any potential bias | This study has certain limitations. Data were collected from a single hospital over a four-month period using consecutive sampling, which may limit generalizability. Important variables, including surgeon experience, preoperative hospital stay, wound drainage, and adherence to aseptic technique, were not included in the analysis, potentially introducing residual confounding (Discussion section, page 21, line no 426 up to 429). |
| Interpretation | 20 | Give a cautious overall interpretation of results considering objectives, limitations, multiplicity of analyses, results from similar studies, and other relevant evidence | In the present study, most patients were admitted to the general surgery ward (59.4%), consistent with findings from India (62%) (25) and Nekemte Hospital, Ethiopia (60.1%) (26). In contrast, higher proportions were observed in a multicenter survey from Ghana (71.5%) (27), whereas a lower proportion was reported at Saint Paul Hospital, Addis Ababa (36.8%) (28), likely reflecting differences in hospital structure and the availability of specialized surgical wards.  Regarding surgical prophylaxis, 69.3% of patients received preoperative antibiotics, similar to reports from the Netherlands (70%) (29), India (67%) (25), and Black Lion Specialized Hospital, Ethiopia (68.7%) (30). Nonetheless, a higher prevalence was documented at Hiwot Fana Comprehensive Specialized Hospital, Ethiopia (86.2%) (31), suggesting institutional differences in prophylaxis protocols and adherence to standard guidelines. Ceftriaxone was the most commonly used preoperative antibiotic (50.2%), followed by ceftriaxone plus metronidazole (27.4%). This differs from findings in Northwest Iran, where cefazolin was utilized in 90% of cases (32). However, comparable prescribing patterns have been reported at Dessie Referral Hospital, Northeast Ethiopia, where 53.6% of patients received ceftriaxone (20), indicating consistency with local practice.  Among patients receiving postoperative antibiotics, 78.3% were prescribed for prophylaxis and 21.7% for treatment. Globally, high rates of postoperative prophylaxis have been reported, including 92% in Italy (33), 84.2% in Bangladesh (34), and 77.5% in India (35). Similar patterns have been observed in Tanzania, where 79.2% of patients received postoperative antibiotics, predominantly ceftriaxone alone (85.7%) (36). In this study, ceftriaxone plus metronidazole was the predominant postoperative regimen (69.6%), in line with findings from Saint Paul Hospital, Addis Ababa (72%) (28). These findings suggest that ceftriaxone-based regimens remain widely used despite guideline recommendations favoring first-generation cephalosporins.  Evaluation of SAP practices showed that 69.3% of patients had an appropriate indication and received prophylactic antibiotics. By comparison, 28.9% had no indication and appropriately did not receive prophylaxis, while 1.8% received prophylactic antibiotics despite the absence of an indication. Previous studies have reported wide variability in inappropriate prophylactic antibiotic use, with rates as high as 44% in Iran and 35.7% in Italy (37,38). Whereas, at Black Lion Specialized Hospital, Ethiopia, 87.4% appropriate use and 12.6% unnecessary administration were reported (39). These findings indicate that inappropriate prophylactic antibiotic use persists across healthcare settings. Ceftriaxone was the most frequently used agent for surgical prophylaxis (50.2%), despite guideline recommendations favoring cefazolin as the first-line option for most surgical procedures. These findings highlight the urgent need for antimicrobial stewardship interventions, including strengthened guideline implementation and targeted prescriber education, to optimize surgical outcomes.  Improper use of prophylactic antibiotics increases the risk of antimicrobial resistance, adverse drug reactions, unnecessary healthcare costs, and SSIs (40). In our cohort, only 16.9% of patients received appropriate antibiotics for surgical prophylaxis. Globally, the appropriateness of SAP varies widely, with appropriate use documented in 70.3%–95% of procedures and inappropriate use ranging from 2.3% to 100% (41). Compared with the 22.3% rate observed at Tibebe Ghion Specialized Hospital, Ethiopia (42), our findings demonstrate poor compliance with recommended surgical prophylaxis practices.  Timely administration of prophylactic antibiotics is essential for SSI prevention, ideally within one hour before incision or up to two hours for agents requiring longer infusion (43). In our study, 77.6% of patients received SAP within one hour prior to incision. Relative to this, a review of 14 global studies showed adherence rates ranging from 12.7% to 100% (41), with Dutch hospitals showing 51% (29) and 90.2% adherence observed at Saint Paul Hospital, Ethiopia (28).  The recommended duration of SAP is generally within 24 hours, with a single pre-incision dose often sufficient (44). In this analysis, 38.5% of patients received prophylaxis for less than 24 hours, compared with 56% documented in Bangalore, India (45), suggesting inadequate adherence to standard guidelines. Regarding dosing, 97.9% of patients received the correct dose, similar to compliance observed at Razi Hospital, Iran (91.8%) , and Saint Paul Hospital, Ethiopia (100%) (28,37). Additionally, all patients received SAP via the recommended route, aligning with findings from India (46), Pakistan (47), and Ethiopia (23), reflecting strong adherence to ASHP guidelines.  SSI risk is influenced by surgery type, patient health, and quality of care, and it is a recognized global health problem (48,49). According to multiple studies, prevalence varies widely, ranging from 2.5% to 41.9% (50). In our study, SSI occurred in 10.8% of patients, comparable to 9.02% at Zewditu Memorial Hospital (51) but lower than rates reported at Finote Selam General Hospital (19.6%), Felege Hiwot Referral Hospital (14.4%), and in Kenya (22%) (52–54). These variations could likely reveal differences in surgical case mix, wound contamination profiles, and patient comorbidities.  **4.2. Possible biological or operational explanations**  The study was conducted at a single public hospital for four months using consecutive sampling. This approach was chosen due to resource constraints, the feasibility of close supervision, the availability of trained data collectors, and the high surgical load during the study period. Conducting the study at a single site also ensured consistency in clinical practice, simplified ethical approvals, and allowed timely completion of data collection, while including all eligible patients minimized selection bias.  In the study setting, ceftriaxone alone or in combination with metronidazole was the most frequently used antibiotic for surgical prophylaxis, instead of the guideline-recommended cefazolin. Limited availability of cefazolin, lack of locally adapted SAP guidelines, habitual broad-spectrum prescribing, prescriber knowledge gaps, and institutional norms may likely contribute to this practice.  Our analysis identified several factors independently associated with SSIs. Elective surgeries were associated with a 43% lower risk compared with emergency procedures, likely due to more timely SAP administration and a lower likelihood of involving contaminated or dirty wounds. Patients without comorbidities were 87% less likely to develop SSIs than those with comorbidities. This may be explained by intact immune function, better tissue perfusion, and faster wound healing. In addition, non-comorbid individuals are less likely to require prolonged procedures, invasive devices, or immunosuppressive medications, all of which can increase the risk of infection in patients with comorbidities.  Prolonged surgical duration was associated with a significantly higher risk of SSIs, with procedures lasting 2–3 hours and over 3 hours showing 4.2 and 5.3-fold increased risk, respectively. This may be attributed to greater tissue trauma and microbial exposure, consistent with global evidence (56) and several Ethiopian studies (20,57,58). Similarly, contaminated and dirty wounds had 3.4- and 9.3-fold higher risk compared to clean wounds, aligned with a study linking wound classification to SSIs (59). These findings are likely due to high microbial load, tissue devitalization, impaired host defenses, and reduced efficacy of prophylactic antibiotics.  SAP practices were also associated with SSI development. Appropriate SAP reduced SSI risk by 33%, in concordance with studies from Egypt and Ethiopia (40,57,60). Conversely, patients receiving SAP more than 1–2 hours before incision had a 5.3-fold higher risk than those who received it within 30 minutes, aligning with global and Ethiopian studies reporting increased SSI risk when antibiotics are administered too early (52,61,62). This likely reflects suboptimal antibiotic concentrations at the time of incision, thereby reducing prophylactic effectiveness and increasing susceptibility to bacterial contamination. Studies confirmed that administering SAP too early leads to low antibiotic levels at the time of incision (63). Similarly, prolonged postoperative SAP (>72 hours) was associated with a 5.6-fold increase in SSI risk, potentially due to the emergence of resistant organisms and subsequent secondary infections (64).  **4.3. Implications for practice and policy**  This study highlights significant gaps between current SAP practices at DTCSH and guideline recommendations. To reduce SSIs, surgical teams should adhere to recommended antibiotic selection, timing, and duration, while hospitals should ensure consistent access to essential drugs such as cefazolin and actively involve clinical pharmacists in perioperative care. Key patient and procedure related factors, including comorbidities, wound type, and duration of surgery, should be considered during surgical planning. Health facilities should strengthen perioperative documentation, implement antimicrobial stewardship programs, and routinely monitor SSI rates. Policymakers should promote adoption of standardized SAP protocols, establish monitoring systems, and support ongoing training of surgical teams in SAP best practices and SSI prevention.  **4.4. Limitations and future directions for research**  This study has certain limitations. Data were collected from a single hospital over a four-month period using consecutive sampling, which may limit generalizability. Important variables, including surgeon experience, preoperative hospital stay, wound drainage, and adherence to aseptic technique, were not included in the analysis, potentially introducing residual confounding. Future studies should adopt multicenter designs, longer follow-up periods, probability-based sampling, and broader variable assessment to strengthen the robustness of the findings (**Discussion section, page no 17 up to 21**). |
| Generalizability | 21 | Discuss the generalizability (external validity) of the study results | Future studies should adopt multicenter designs, longer follow-up periods, probability-based sampling, and broader variable assessment to strengthen the robustness of the findings (**Discussion section, page 21, line no 430 & 431**). |
| Other information | | | |
| Funding | 22 | Give the source of funding and the role of the funders for the present study and, if applicable, for the original study on which the present article is based | The author declares that the research, writing, and publication of this article were carried out without external financial support (**Page 22, line no 469 & 470**). |

**Note:** An Explanation and Elaboration article discusses each checklist item and gives methodological background and published examples of transparent reporting. The STROBE checklist is best used in conjunction with this article (freely available on the Web sites of PLoS Medicine at http://www.plosmedicine.org/, Annals of Internal Medicine at http://www.annals.org/, and Epidemiology at http://www.epidem.com/). Information on the STROBE Initiative is available at www.strobe-statement.org.
